# Thymic-Epithelial-Cell-Dependent Microenvironment Influences Proliferation and Apoptosis of Leukemic Cells

**DOI:** 10.3390/ijms25031412

**Published:** 2024-01-24

**Authors:** Sandesh Kumar Patel, Nadezda Zhdanovskaya, Ilaria Sergio, Antonella Cardinale, Marco Rosichini, Claudia Varricchio, Eleonora Pace, Carlo Capalbo, Franco Locatelli, Alberto Macone, Enrico Velardi, Rocco Palermo, Maria Pia Felli

**Affiliations:** 1 Department of Molecular Medicine, Sapienza University of Rome, 00161 Roma, Italy; sandeshkumar.patel@uniroma1.it (S.K.P.); nadezda.zhdanovskaya@uniroma1.it (N.Z.); claudia.varricchio@uniroma1.it (C.V.); eleonora.pace@uniroma1.it (E.P.); carlo.capalbo@uniroma1.it (C.C.); rocco.palermo@uniroma1.it (R.P.); 2 Department of Experimental Medicine, Sapienza University of Rome, 00161 Roma, Italy; ilaria.sergio@uniroma1.it; 3 Research Area of Hematology and Oncology, Cell and Gene Therapy, Bambino Gesù Children’s Hospital, IRCCS, 00146 Rome, Italy; antonella.cardinale@opbg.net (A.C.); marco.rosichini@opbg.net (M.R.); franco.locatelli@opbg.net (F.L.); enrico.velardi@opbg.net (E.V.); 4 Department of Life Sciences and Public Health, Catholic University of the Sacred Heart, 12631 Rome, Italy; 5 Department of Biochemical Sciences “A. Rossi Fanelli”, Sapienza University of Rome, 00161 Roma, Italy; alberto.macone@uniroma1.it

**Keywords:** NOTCH, thymic epithelial cells, T-cell leukemia, lympho–stromal crosstalk

## Abstract

T-cell acute lymphoblastic leukemia (T-ALL) is a hematological cancer characterized by the infiltration of immature T-cells in the bone marrow. Aberrant NOTCH signaling in T-ALL is mainly triggered by activating mutations of NOTCH1 and overexpression of NOTCH3, and rarely is it linked to NOTCH3-activating mutations. Besides the known critical role of NOTCH, the nature of intrathymic microenvironment-dependent mechanisms able to render immature thymocytes, presumably pre-leukemic cells, capable of escaping thymus retention and infiltrating the bone marrow is still unclear. An important challenge is understanding how leukemic cells shape their tumor microenvironment to increase their ability to infiltrate and survive within. Our previous data indicated that hyperactive NOTCH3 affects the CXCL12/CXCR4 system and may interfere with T-cell/stroma interactions within the thymus. This study aims to identify the biological effects of the reciprocal interactions between human leukemic cell lines and thymic epithelial cell (TEC)-derived soluble factors in modulating NOTCH signaling and survival programs of T-ALL cells and TECs. The overarching hypothesis is that this crosstalk can influence the progressive stages of T-cell development driving T-cell leukemia. Thus, we investigated the effect of extracellular space conditioned by T-ALL cell lines (Jurkat, TALL1, and Loucy) and TECs and studied their reciprocal regulation of cell cycle and survival. In support, we also detected metabolic changes as potential drivers of leukemic cell survival. Our studies could shed light on T-cell/stroma crosstalk to human leukemic cells and propose our culture system to test pharmacological treatment for T-ALL.

## 1. Introduction

T-cell acute lymphoblastic leukemia (T-ALL) is an aggressive blood cancer arising from the malignant transformation and clonal expansion of T-cell progenitors, mainly occurring in children and adolescents and rarely in older age [1,2]. Despite current frontline standard care results in high overall survival in most pediatric T-ALL cases, treatments in relapsed or refractory patients remain challenging due to the limited number of additional therapeutic interventions available [3]. A better understanding of the molecular mechanisms driving the disruption of the T-cell developmental program and T-ALL progression will guide designing innovative interventions and novel therapeutic targets for treating this malignancy. T-ALL arises from the progressive accumulation of genetic and epigenetic alterations in critical genes involved in the physiological thymocyte development [3]. Given the prominent role of NOTCH signaling in T-cell differentiation and fate specification [4,5], the aberrant activity of its pathway is strictly linked to T-ALL onset and progression. Consistently, approximately half of T-ALL cases are associated with enhanced NOTCH signaling, and patient leukemic cells frequently bear somatic gain-of-function gene mutations in *NOTCH1* and/or inactivating mutations of the NOTCH negative regulator *FBXW7* [6,7]. Moreover, rare cases of *NOTCH3*-activating mutations have been described in T-ALL patients [8]. In recent decades, much effort has been dedicated to elucidating genes and pathways controlled by NOTCH and contributing to malignant lymphoblasts’ aberrant survival and proliferation. However, the nature of the immature T-cells escaping thymus retention and infiltrating the bone marrow remains unclear. These cells could represent leukemia-initiating cells, and the mechanisms underlying their dissemination need to be clarified.

T-ALL originates in the thymus and is characterized by bone marrow infiltration by leukemic blasts and is frequently associated with the involvement of other organs like the central nervous system, the mediastinal lymphoid organs, and the spleen [9,10]. Enhanced leukemia cell homing and infiltration are related to the modulation of T-ALL metabolic activity operated by microRNA and overexpressed genes [11,12]. Indeed, besides acquiring molecular signatures driving the uncontrollable proliferation of immature T-cell precursors, T-ALL cells require stromal cell signals to survive, and bone marrow [13] and thymic microenvironments participate in this crosstalk [14]. A critical challenge for designing novel T-ALL treatment approaches is understanding how leukemic cells shape the tumor microenvironment to increase their ability to infiltrate and survive within.

Thymic epithelial cells (TECs) represent the most abundant population of thymic stroma and, in physiological conditions, are known to promote survival and maturation of maturating thymocytes [15,16], driving both positive and negative selection through a multitude of signaling pathways, including SDF-1/CXCR4, IL-7/IL-7R, PI3K, SHH, and NOTCH signaling [17,18,19,20,21]. While it is well known that bone marrow homing is orchestrated by the SDF-1/CXCR4 axis [22,23,24], thymic infiltration appears to be less dependent on this pathway [10]. However, SDF-1 is produced by TEC cells, and the SDF-1/CXCR4 axis confers survival and expansion signals to early T-cell progenitors [25]. In addition, several NOTCH ligands are expressed by TECs (such as DLL1, DLL4, JAG1, and JAG2), and the interaction between NOTCH1 receptors on the surface of immature T-cells and TECs is known to induce T-cell commitment. However, only DLL4 is essential during thymic T-cell development [26,27,28,29,30]. Accordingly, interaction with stromal stimuli like SDF-1 or NOTCH ligands provides an essential survival signal for T-ALL cells otherwise predisposed to apoptotic cell death [10,31]. Previously, we demonstrated that overexpression of NOTCH3 reduced the spontaneous apoptosis rate in SDF-1-treated immature thymocytes [14]. The interaction between bone marrow stroma and leukemic cells [13,22] and the role of the thymic microenvironment in thymocyte maturation have been extensively studied [13,22,29]. On the other hand, little is known about the interactions occurring between TEC and T-cell lymphoblasts, with few studies evidencing that TEC creates favorable conditions for leukemic cell expansion through the aberrant activity of transcriptional factors like FoxN1 and soluble mediators like IL-7 [32,33]. The implications of NOTCH signaling in the crosstalk between TEC and leukemic cells, along with the precise role of different NOTCH paralogs and ligands in mediating pro-survival, proapoptotic, pro-differentiation, pro-migratory and metabolic signals, need a more detailed description.

In this pilot study, we aimed to shed light on the crosstalk between thymic epithelial cells and human leukemic cells and to identify the effect of the reciprocal interactions in modulating NOTCH signaling, metabolism, cell cycle progression, and viability that might influence the progressive disruption of T-cell development driving T-cell leukemia.

## 2. Results

### 2.1. TEC-Conditioned Medium Provides a Microenvironment Influencing T-ALL Cell Viability

**Figure 1:**
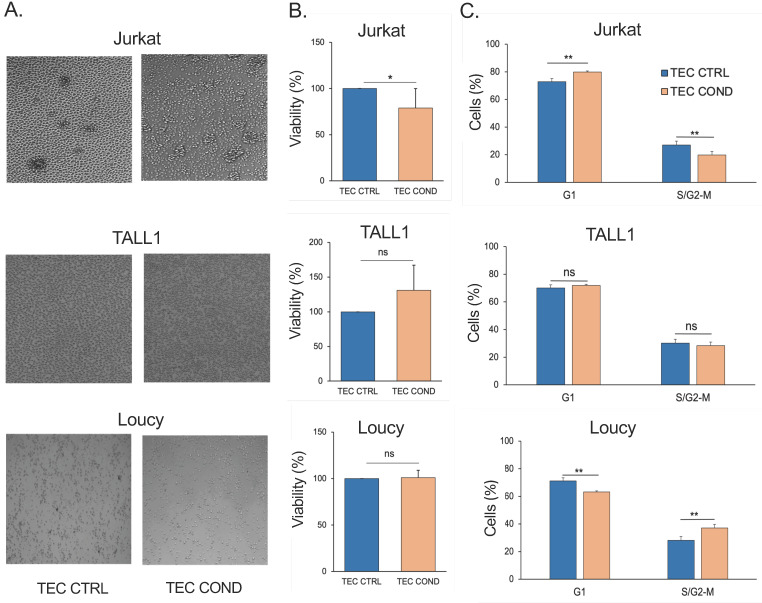
Differential T-ALL cell lines viability in the TEC-conditioned culture system. (**A**) Representative optical microscopic images (EVOS) 24 h post-incubation of Jurkat, TALL1, and Loucy cells in the medium used to cultivate hTECs (TEC CTRL) and medium conditioned by 48 h hTECs culture (TEC COND). The magnification of the images is 10×. (**B**) The percentages of the viable Jurkat, TALL1, and Loucy cells by trypan exclusion test in the different conditioned media. (**C**) Quantitative cell cycle analysis of PI-stained cells 24 h post-incubation with TEC CTRL and TEC COND. G1 and S/G2–M are represented for each condition. Histograms represent the mean of four biological replicates ± standard deviation (SD) from four independent experiments. Statistics were performed with Student’s *t*-test in the two different culture conditions (* *p* < 0.05; ** *p* < 0.01; ns, not significant).

We first hypothesized that hTECs, freshly isolated from the human thymus, could release multiple soluble factors that typically commit T-cell development but that could either sustain or restrain human T-ALL cell lines’ viability and growth. Therefore, we investigated the effects of primary human TEC conditioned medium (TEC COND) on NOTCH1-dependent Jurkat, NOTCH3-dependent TALL1, or NOTCH-independent Loucy leukemic T-cell lines. After 24 h of culture, in human TEC (TEC CTRL) or with a cell-free TEC COND medium, the biological parameters of T lymphoblast Jurkat and the more immature TALL1 and early T-cell progenitor (ETP) Loucy cells were analyzed. No morphological changes were found in all cell lines under the influence of the TEC COND medium. However, 24 h post-incubation, Jurkat cell density decreased in TEC COND compared to the human TEC CTRL medium (Figure 1A). Conversely, TALL1 and Loucy cells grew scattered without any evident change (Figure 1A). These observations may suggest a different adaptability of human T-ALL cell lines to the microenvironment imprinted by the hTECs. To this end, we evaluated the percentage of live cells 24 h post-incubation by trypan blue exclusion test of cell viability. Resembling the microscope observations, Jurkat cells displayed a reduction in the percentage of living cells from the TEC CTRL to the TEC COND media, thus suggesting that although being malignant and glucocorticoid-resistant [34], Jurkat cells are still sensitive to stimuli and growth control triggered by physiological primary hTECs. We could hypothesize that the TEC COND medium contains or lacks factors that modulate the viability of this leukemic cell line. On the other hand, TALL1 and Loucy cell lines were not susceptible to the TEC COND medium (Figure 1B).

We further addressed the impact of the different culture conditions on the cell cycle progression (G1, S/G2–M phases) of the three cell lines. Data of flow cytometric analysis in Figure 1C documented that the TEC COND medium induces the substantial accumulation of Jurkat cells in G1 and a consequent decrease in cells in the S/G2–M phases, thus supporting a G1 cell cycle arrest. Conversely, TALL1 cells grown in the TEC COND medium do not display any significant change in cell distribution within the distinct cell cycle phases compared to the TEC CTRL counterpart growth (Figure 1C). Conversely, in the TEC COND medium, we observed an altered cell cycle phase distribution in Loucy cells, with a shift from the G1 to the S/G2–M phase as compared to TEC CTRL (Figure 1). The percentage of Loucy cells in the S phase slightly increased in the TEC COND medium.

Overall, our data strongly suggest that hTECs can influence Jurkat T-ALL cell lines’ viability and cell cycle by conditioning the extracellular microenvironment more suitable for TALL1 and Loucy cell growth.

### 2.2. Jurkat, TALL1, and Loucy Cells Differ in Their Biological and Metabolic Profile when Cultured in a TEC-Conditioned Medium

Considering the observed effects of the human TEC-conditioned medium (Figure 1), we further analyzed the apoptotic rate of Jurkat, TALL1, and Loucy cell lines in this culture system.

**Figure 2:**
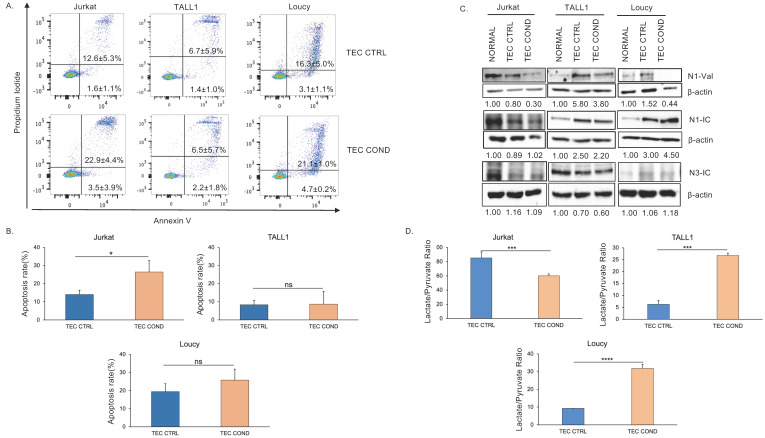
TEC-conditioned medium differently modulates NOTCH signaling and the metabolic profiles of T-ALL cell lines. A possible correlation to apoptosis. Jurkat, TALL1, and Loucy cells FACS analysis 24 h post-incubation: (**A**) FACS analysis with propidium iodide and annexin V to evaluate apoptotic cells. The percentages of early (bottom-right quadrant) and late (upper-right quadrant) apoptotic cells are indicated and represent the mean ± standard deviation (SD) of four biological replicates. (**B**) Statistical evaluation of apoptotic rates, including early and late apoptotic cells, in each cell line and condition. Histograms represent the mean of four biological replicates ± SD. Statistics were performed with Student’s t-test in the two different culture conditions (*p*-value < 0.05 *; ns, not significant). (**C**) Representative Western blots analysis of active NOTCH1 (N1-val), intracellular NOTCH1 domain (N1-IC), and active intracellular NOTCH3 domain (N3IC), proteins quantification by densitometric analysis, normalized to *β*-actin (fold induction) of three independent experiments. (**D**) Lactate/pyruvate ratio normalized to internal control in the three culture conditions following 24 h post-incubation. Histograms represent the mean of four biological replicates ± SD from four independent experiments. Statistics were performed with Student’s t-test in the two different culture conditions (* *p* < 0.05; *** *p* < 0.001; **** *p* < 0.0001; ns, not significant).

In keeping with the viability and the cell cycle data of Jurkat cells (Figure 1B,C), the combined staining with propidium iodide (PI) and annexin V (Figure 2A), 24 h post-incubation further supported the reduced growth of Jurkat cells in TEC COND as compared to TEC CTRL. Indeed, in TEC COND, both the early and late apoptotic Jurkat cells increase compared to the TEC CTRL condition. In support, the percentage of apoptotic cells is statistically increased in Jurkat grown in TEC COND versus TEC CTRL (Figure 2B). In contrast, PI/annexin V staining revealed a comparable frequency of the early and late apoptotic TALL1 cells in TEC COND and in the TEC CTRL medium (Figure 2A,B) in agreement with data shown in Figure 1B,C. Indeed, for TALL1, the rate of apoptosis is similar in TEC CTRL versus TEC COND media (Figure 2B). It is worth mentioning that the high apoptotic rate in Loucy cells in the TEC CTRL medium is comparable with the apoptotic rate in the standard culture medium.

To correlate NOTCH expression to TEC COND effects, we monitored NOTCH1 and NOTCH3 protein expression in all three cell lines, including the NOTCH-independent Loucy cells. We investigated NOTCH signaling modulation measured by the expression level of endogenous activated/intracellular domains following 24 h exposure to the TEC-conditioned medium (Figure 2C).

Of note, Jurkat and TALL1 cell lines express active forms of distinct NOTCH receptors at the basal level (normal), with Jurkat characterized by NOTCH1 signaling activation, whereas TALL1 by NOTCH3. In Jurkat, the active NOTCH1 (N1-Val) is decreased in TEC COND versus TEC CTRL, whereas no change is observed for the intracellular form of NOTCH1 and NOTCH3 (N1-IC and N3-IC), in support of a reduced signaling activation. Inversely, TALL1 cells express N3-IC, and although TEC CTRL surprisingly increased N1-Val and N1-IC, they are slightly downregulated in the TEC COND setting (Figure 2C). On the other hand, despite N1-IC and N3-IC being nearly undetected in Loucy cells, unexpectedly, N1-IC increased in TEC CTRL and persisted in TEC COND medium (Figure 2C). These data, in association with the percentages of early and late apoptotic cells (Figure 2A,B), may suggest that the downmodulation of the active NOTCH1 correlates with the enhanced Jurkat cell death in the TEC COND medium. Indeed, overactive NOTCH signaling is involved in the regulatory networks that drive T-ALL growth and survival [35].

It has been reported that the extracellular lactate/pyruvate ratio modulates the sensitivity to a hepatoma cell line’s oxidative-stress-induced apoptosis via the cytosolic NADH/NAD^+^ redox state [36]. Interestingly, we found that TEC COND medium triggered at 24 h a substantial decrease in the extracellular lactate/pyruvate ratio in comparison to TEC CTRL in Jurkat cells (Figure 2D). Altogether, these results argue in favor of a proapoptotic effect in Jurkat cells following 24 h exposure to the TEC-conditioned medium, which presumably provides components that reduce the adaptation ability of more mature T lymphoblasts.

Additionally, TALL1 cells cultured with TEC COND medium displayed a different metabolic profile at 24 h with a higher lactate/pyruvate ratio when compared to TEC CTRL (Figure 2D). Therefore, dynamic metabolic changes differently characterize Jurkat and TALL1 cells in a TEC COND medium and can be correlated to apoptosis. Within 24 h of culture, the more immature TALL1 cells adapt, and the increased extracellular lactate/pyruvate ratio may correlate with the slight, despite not significant, increase in cell viability and no changes in cell cycle distribution (Figure 1B) and the apoptotic rate (Figure 2A,B) in TEC COND versus TEC CTRL media. Interestingly, NOTCH-independent Loucy cells with no changes in cell viability but a shift of cell percentages from the G1 to the S/G2–M phase (Figure 1B,C) and a high but constant apoptotic rate (Figure 2A,B) display a higher lactate-to-pyruvate ratio in TEC COND versus TEC CTRL (Figure 2D). Overall, our data propose that malignant T-cell lines have different adaptation abilities in response to the apoptotic stress induced by a TEC COND medium, possibly relying on NOTCH1 signaling downmodulation. Furthermore, the data of NOTCH-independent Loucy cells, having an early T-precursors (ETP)-ALL phenotype [37], may suggest that immature T-ALL cells are less prone to growth arrest when in a medium conditioned by healthy TECs.

### 2.3. Leukemic Cells Conditioned Medium Regulates Survival and Gene Expression of TECs

**Figure 3:**
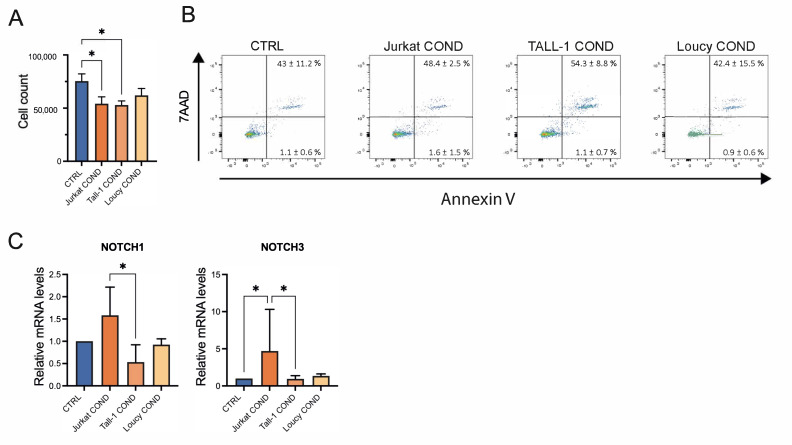
Modulation of hTEC survival and NOTCH signaling by T-ALL cell lines conditioned medium. (**A**) Analysis of cell count by Trypan blue exclusion assays of TEC in different culture conditions. Histograms represent the mean of four replicates obtained with two biological samples ± standard deviation (SD) from two independent experiments. (**B**) FACS analysis with 7AAD and annexin V staining to evaluate apoptotic cells 24 h post-incubation. The early (bottom-right quadrant) and late (upper-right quadrant) percentages of apoptotic cells are indicated. FACS plots are representative of two independent experiments obtained with two biological replicates. (**C**) Evaluation of *NOTCH1* and *NOTCH3* gene expression levels at 24 h post-exposure to the Jurkat-, TALL1-, or Loucy-COND medium by qPCR. Histograms represent the mean of four to eight replicates obtained with two to four biological samples ± SD from four independent experiments. Statistics were performed with the Mann–Whitney nonparametric test (* *p* < 0.05; ns, not significant).

Crosstalk between TECs and cells of hematopoietic origin is crucial for correct maturation, differentiation, and function of thymic epithelium. Thus, we investigated whether soluble factors released by T-ALL cells could impact hTEC survival and expression of key factors crucial for epithelial cell development and function. To study this, we exposed primary hTECs to the T-ALL cells COND medium. In the evaluation of total cell number after incubation with Jurkat-, TALL1-, or Loucy-COND, we found that hTECs displayed morphological changes and reduced growth, particularly when cultured with Jurkat and TALL1 COND medium, as compared to cells grown in T-ALL control (CTRL) medium (Figure 3A). These data were confirmed by annexin V/7AAD staining, which showed increased apoptotic cells in the Jurkat and TALL1 COND medium group versus the CTRL medium counterpart (Figure 3B). To further investigate the impact of the T-ALL conditioned medium on hTECs, we assessed the expression of NOTCH1, NOTCH3, DLL1, and DLL4, key genes regulating TEC function. While no major differences were observed in the expression of DLL1 and DLL4, we detected significant upregulation of NOTCH3 and trends toward higher levels of NOTCH1 in hTEC after 24 h post-exposure with the Jurkat COND medium (Figure 3C). These data suggest that Jurkat cells release soluble factors that regulate the survival and gene expression of hTECs.

## 3. Discussion

Our results indicate that the hTEC-conditioned microenvironment of our culture medium modulates the NOTCH signaling and the biological programs of human T-ALL cell lines, Jurkat, and freshly isolated hTEC cells with a milder effect on the immature TALL1 and ETP-ALL Loucy cells.

The culture with the TEC COND medium affects the growth and apoptosis of Jurkat, TALL1, and Loucy cells differently. Indeed, Jurkat cells, with a mature and glucocorticoid-resistant phenotype [34], can grow in the specific medium used for hTECs, which contains hydrocortisone. In contrast, the more immature TALL1 cells greatly suffer in the TEC CTRL medium. Interestingly, Jurkat cells became sensitive to apoptosis when exposed to the TEC COND medium, in which active NOTCH1 (N1-Val) downmodulation impinges on survival pathways. This evidence suggests that a healthy thymic microenvironment can still modulate T-ALL cell growth and commit a leukemic cell to death. Therefore, new studies will help to better characterize the factor/s involved.

Conversely, we hypothesize that the more immature NOTCH1-independent TALL1 cells, overexpressing the active N3-IC, can better adapt to stress conditions in the TEC COND medium, possibly reprogramming the metabolic pathways to suit the survival needs. Indeed, the different metabolic programs of mature Jurkat and immature TALL1 cells can match expansion demands and could be correlated to metabolic alterations reported during the development of a T-cell, possibly relying on the balance into equilibrating activation programs and metabolic quiescence [38]. Additionally, the ETP-ALL cell line Loucy, which shows a transcriptional program related to immature T-ALL [39], does not display any correlation between NOTCH signaling, growth, and apoptosis.

Cancer cells manage various biological processes to ensure survival and proliferation, even under unfavorable conditions. It has been recently suggested that this effect relies on ‘metabolic reservoirs’ [40], which represent a pool of vital metabolites ready to be used by cancer cells. It has been reported that the Warburg effect in cancer cells generates an anti-apoptotic extracellular microenvironment by elevating the extracellular lactate/pyruvate ratio, which desensitizes HepG2 cells toward apoptotic insult [41]. Indeed, dynamic metabolic changes characterize the heterogenous responses of Jurkat, TALL1, and the NOTCH-independent Loucy cells under a TEC-conditioned medium. Jurkat cells might be able to rely more on glycolysis due to PTEN mutations [42] when NOTCH signaling is inhibited as in TEC CTRL medium; however, they have poor adaptability and die in keeping with the decreased lactate/pyruvate ratio observed following 24 h incubation in a TEC-conditioned microenvironment. In that context, TALL1, not harboring PTEN mutations, cells slowly increase the extracellular lactate/pyruvate ratio, which correlates with the decreased percentages of early and late apoptotic cells concerning the TEC CTRL medium. Loucy cells sensible to the TEC COND microenvironment display a proliferative and metabolic profile supporting a NOTCH-independent survival program.

Our preliminary results suggest a correlation between the kinetic of the extracellular lactate-to-pyruvate ratio and apoptosis in T-ALL cell lines. The molecular and biochemical mechanisms of glycolysis in T-ALL remain largely unexplored. Cell migration is an energy-demanding process, so we speculate that metabolic targeting can also affect CXCR4-mediated T-ALL migration, as recently suggested [11]. Further studies with our culture system may uncover novel ways for metabolic targeting in T-ALL.

On the contrary, our study on the impact of leukemic Jurkat, TALL1, and Loucy cells on hTECs revealed that they modulate the survival and gene expression of a normal thymic epithelium. Of note, while we observed a negative impact on cell growth and survival, the Jurkat COND medium induced increased expression of NOTCH receptors and, particularly, of NOTCH3 in hTECs.

Recent studies extended the role of NOTCH signaling to TECs, demonstrating that NOTCH regulates TEC homeostasis in fetal and adult life [16,43]. NOTCH1 plays key roles in TEC progenitor maintenance and the maturation of medullary TECs. In addition, forced NOTCH activation in TECs negatively influences TEC differentiation and affects the normal process of T-cell development [40]. The increased expression of NOTCH3 in this study, after Jurkat COND medium treatment, may suggest that activation of NOTCH signaling can induce a detrimental effect on TECs in vitro differentiation and expansion or directly activate apoptosis, as reported by studies in other cellular models [44].

We are aware that our in vitro model still has certain limitations. This study focused on the effect of TEC- and T-ALL-imprinted microenvironments on the viability, cell cycle progression, and apoptosis of T-cell leukemia cell lines and the TECs, respectively. However, the different conditioned media are remarkably complex in terms of biochemical conditions and might include a variety of active factors potentially released by cells. Our studies did not identify the most influential one from the abundant soluble factors in the media. Still, they could inform how the conditioned extracellular space is sufficient per se to modulate some biological parameters in the exposed cells. Nonetheless, our studies propose a novel cell culture model that could be helpful in testing the efficacy of drugs and hopefully designing cancer-metabolism-targeting strategies to achieve synergy with traditional therapy and better results.

## 4. Materials and Methods

### 4.1. Cell Culture and Generation of Human TEC Culture

TALL1, Jurkat E6-1 (kindly provided by academic colleagues), and Loucy (purchased from the ATCC, #CRL-2629, Manassas, VA, USA) cells were cultured at 37 °C and 5% CO_2_ in a complete medium, RPMI 1640, supplemented with 15% FBS, 10 U/mL penicillin–streptomycin, and 2 mM L-glutamine.

Cell culture of primary human thymic epithelial cells (hTECs) was established following the protocol originally described by Green et al. with modifications [45]. Thymic tissues were obtained during corrective cardiovascular surgery of pediatric subjects. The thymus capsule was removed, and thymic specimens were finely minced and suspended in RPMI medium (Euroclone, Pero, Italy) supplemented with 0.06 mg/mL Liberase (Merck, Darmstadt, Germany), 2 mM L-glutamine (Euroclone, Pero, Italy), and 0.4 mg/mL DNAse from bovine pancreas (Roche, Basel, Switzerland). Three digestion cycles (20 min each) were performed at 37 °C in agitation. Digested samples were collected in RPMI containing 2% FBS (ThermoFisher, Waltham, MA, USA), pooled, and spun at 300 g for 5 min at 4 °C. For TEC selection, epithelial cell adhesion molecule (EpCAM)-positive cells were enriched using anti-EpCAM (CD326) (clone HEA-125, Miltenyi, Bergisch Gladbach, Germany) staining and anti-APC microbeads (Miltenyi, Bergisch Gladbach, Germany) magnetic separation. The TEC-enriched cell fraction was plated (2.5 × 10^4^/cm^2^) on irradiated 3T3-J2 murine fibroblasts (Kerafast, Boston, MA, USA) acting as a feeder layer. Cells were cultured in a humidified atmosphere of 5% CO_2_ in a medium composed of DMEM:Ham’s F-12 (3:1 ratio), 10% fetal calf serum (FCS) (ThermoFisher, Waltham, MA, USA), adenine (0.18 mM, Sigma-Aldrich, St. Louis, MO, USA), hydrocortisone (0.4 μg/mL, Sigma-Aldrich, St. Louis, MO, USA), insulin (5 μg/mL, Eli Lilly, Indianapolis, Indiana), cholera toxin (0.1 nM, List Labs, Campbell, CA, USA), glutamine (4 mM, ThermoFisher, Waltham, MA, USA), triiodothyronine (2 nM, Sigma), and antibiotics. Epidermal growth factor (10 ng/mL, Austral Biologicals, San Ramon, CA, USA) was added to the medium after 48 h of culture. Cultured TECs were assessed by standard light-sheet microscopy for the maintenance of TEC-like morphology and by FACS for the expression of the TEC markers EpCAM, Ulex Europaeus Agglutinin I (UEA-I, clone GoH3, DBA), and CD205 (clone C205, BD, Franklin Lakes, NJ, USA) to discriminate between mTECs (CD205low UEA-high) and cTECs (CD205high UEA-1low). Twenty-four hours before the start of the experiments, hTECs were separated from the feeder layer and plated in KGM-Gold medium (Lonza, Basel, Switzerland). Our TEC culture system preferentially enriches for mTECs [46].

### 4.2. Culture Conditions (Cell Viability and Morphology)

After 48 h Jurkat, TALL1, and Loucy cell lines culture, a cell-free medium, which has been completely depleted of cells, was extracted (Jurkat-, TALL1-, and Loucy-COND) and used to treat thymic epithelial cells for a 24 h culture. T-ALL untreated media (Jurkat-, TALL1-, and Loucy-CTRL media) were used as control. Conversely, Jurkat, TALL1, and Loucy cells were cultivated with hTECs conditioned cell-free medium (TEC COND), hTEC control medium (TEC CTRL), and T-ALL cells standard medium (normal) at 1 million cells/mL. At 24 h, cell viability was analyzed by Trypan blue dye exclusion test. Morphological changes were recorded using live-image microscopy through EVOS coreX1 microscope at 10× magnification.

### 4.3. Western Blot Analysis

Proteins extracted from Jurkat, TALL1, and Loucy cells using sonication were separated by SDS-PAGE, transferred to nitrocellulose membranes, and probed with the following antibodies: NOTCH1Val1744 (#4147), NOTCH3Intracellular (IC) domain (#2889), NOTCH1 IC domain (#3608) all purchased from Cell Signaling Technology, Danvers, MA, USA; and β-actin (A5441; Sigma-Aldrich) [7]. The presence of the bound antibodies was detected using HRP-conjugated secondary antibodies and enhanced chemiluminescence using the Azure imaging system. Images were acquired by Image Lab Software (Bio-Rad), using a ChemiDoc System instrument (Bio-Rad). Densitometric evaluation was performed after normalization using Image J software 1.53m.

### 4.4. Flow Cytometry Analysis (Cell Cycle and Apoptosis)

FACS analysis of Jurkat, TALL1, and Loucy cells, excluding the dead cells, was performed by using a forward- and side-scattering gate technique [14]. To analyze cell cycle distribution, cells were fixed with ice-cold 70% ethanol for 24 h at 4 °C. Next, cells were washed in PBS buffer and stained with 500 μL of PBS containing RNase A (100 μg/mL, Sigma-Aldrich) and propidium iodide (50 μg/mL) for 30 min in the dark. A total of 1 × 10^6^ cells were collected and washed twice in PBS. Cell apoptosis was determined by flow cytometric analysis using annexin V (BD Pharmigen, San Diego, CA, USA, Cat#550474)/propidium iodide (PI) (BD Pharmigen, Cat#556463) or 7AAD (BD Pharmingen, Cat#51-68981E) staining as previously described in [47]. Samples were analyzed on a FACS-Calibur or BD LSRFortessa X20 with CellQuest or FlowJo v10.9 software (BD-Biosciences, San Jose, CA, USA).

### 4.5. Lactate and Pyruvate Assay

Lactic acid and pyruvic acid were analyzed as methoxime/tertbutyldimethylsilyl derivatives as previously described [48]. Briefly, 20 μL of cell-free culture medium was deproteinized by adding 100 μL of acetonitrile and vortexed for 3 min. The mixtures were diluted 1:10 with distilled water and centrifuged at 15,000 rpm for 15 min at 4 °C to pellet proteins. The deproteinized supernatant was used to quantify extracellular L-lactate by GC-SIM-MS analysis. Aliquots of 0.25 mL of the supernatant layer spiked with the internal standard (IS) 3,4-dimethoxybenzoyc acid (final concentration 1000 ng mL^−1^) were added to 0.7 mL of distilled water and adjusted to pH ≥ 13 with 7 M NaOH. Methoxymation was performed by adding to the reaction mix methoxyamine hydrochloride (5 mg) at 60 °C for 60 min. The samples were then washed with diethylether (3 mL × 2), and the aqueous phase was adjusted to pH < 2 with concentrated sulfuric acid. The mixture was saturated with NaCl and extracted with diethyl ether (3 mL) and ethyl acetate (2 mL). The organic extracts were combined in the presence of triethylamine (10 μL) and dried under reduced pressure. The samples were then suspended in 30 μL of toluene and subjected to the second derivatization step by adding 20 mL of MTBSTFA (65 °C for 30 min) and analyzed by GC-MS. Results were normalized by cell number and expressed as fold change relative to control samples.

GC-M analyses were performed with an Agilent 7890B gas chromatograph coupled to a 5977B quadrupole mass selective detector (Agilent Technologies, Palo Alto, CA, USA). Chromatographic separations were carried out with an Agilent HP5ms fused-silica capillary column (30 m × 0.25 mm i.d.) coated with 5% phenyl–95% dimethylpolysiloxane (film thickness 0.25 μm) as stationary phase. Injection mode: splitless at a temperature of 280 °C. Column temperature program: 70 °C (1 min), then to 300 °C at a rate of 20 °C/min and held for 10 min. The carrier gas was helium at a constant flow of 1.0 mL/min. The spectra were obtained in the electron impact mode at 70 eV ionization energy; ion source 280 °C; ion source vacuum 10–5 Torr. MS analysis was performed simultaneously in TIC (mass range scan from *m*/*z* 50 to 600 at a rate of 0.42 scans s^−1^) and SIM mode. GC-SIM-MS analysis was performed by selecting the following ions: *m*/*z* 174 for pyruvate, *m*/*z* 261 for lactate, and *m*/*z* 239 for 3,4-dimethoxybenzoic acid (internal standard).

### 4.6. Gene Expression Analysis

RNA was extracted from hTEC using RNeasy Plus Micro Kit (Qiagen) and retro-transcribed through High-Capacity cDNA Reverse Transcription Kit (Applied Biosystems, Waltham, MA, USA) according to the manufacturer’s instructions. The quantitative polymerase chain reaction (qPCR) standard protocol was used to assess gene expression. Luna Universal Probe Master Mix (Euroclone, Pero, Italy) was used along with the following TaqMan probes (ThermoFisher, Waltham, MA, USA): *DLL1* (Hs00194509_m1), *DLL4* (Hs00184092_m1), *NOTCH1* (Hs01062014_m1), *NOTCH3* (Hs01128537_m1), and *GAPDH* (Hs02786624_g1). qPCR was performed in a QuantStudio 7 Pro (Applied Biosystems, Waltham, MA, USA).

### 4.7. Statistical Analysis

All data are expressed as mean ± SD and percentages of four independent experiments. Statistical significance was performed with Student’s *t*-test to compare two groups or with the Mann–Whitney nonparametric test applied to compare more groups. Statistical analysis was performed using GraphPad Prism Software Version 8.0 (GraphPad Software, Inc.). A *p*-value < 0.05 was considered as significantly different.

### 4.8. Patient Samples

Human thymic samples for hTEC culture were obtained from pediatric patients undergoing corrective cardiac surgery. All samples were anonymously collected in accordance with local ethical guidelines, written informed consent was obtained, and the protocol was approved by the Ethics Committee of OPBG Hospital.

## Data Availability

The data presented in this study are available upon request from the corresponding author.
